# Multi-traction multi-tunneling techniques combined with novel enhanced imaging to facilitate endoscopic submucosal dissection of large rectal lesions

**DOI:** 10.1055/a-2791-5029

**Published:** 2026-02-24

**Authors:** Daryl Ramai, Abdulrahman Qatomah, Daniela Kurfurstova, Premysl Falt, Ondrej Urban, Hiroyuki Aihara, Lumir Kunovsky

**Affiliations:** 1 1861 Division of Gastroenterology, Hepatology and Endoscopy, Brigham and Women’s Hospital, Boston, Massachusetts, United States; 2 14434 Division of Gastroenterology, Hepatology and Nutrition, University of Utah School of Medicine, Salt Lake City, Utah, United States; 3 37852 Department of Medicine, King Faisal Specialist Hospital and Research Center, Jeddah, Saudi Arabia; 4 48233 Institute of Clinical and Molecular Pathology, University Hospital Olomouc, Faculty of Medicine and Dentistry, Palacky University Olomouc, Olomouc, Czech Republic; 5 2nd Department of Internal Medicine – Gastroenterology and Geriatrics, University Hospital Olomouc, Faculty of Medicine and Dentistry, Palacky University Olomouc, Olomouc, Czech Republic; 6 Department of Gastroenterology and Digestive Endoscopy,, Masaryk Memorial Cancer Institute, Brno, Czech Republic; 7 37748 Department of Surgery, University Hospital Brno, Faculty of Medicine, Masaryk University, Brno, Czech Republic

Endoscopic submucosal dissection (ESD) is the preferred treatment for large colorectal lesions due to its ability to achieve en-bloc resection and high curative resection rates 1 2 . Multiple adjunctive techniques, including traction, tunneling, and pocket creation, have been developed to improve technical efficiency and safety 3 . More recently, novel image-enhancement technologies have been introduced to optimize the visualization of submucosal planes and vascular structures during ESD. We report a case demonstrating the combined use of multi-traction, multi-tunneling, and amber-red color imaging (ACI) in the resection of a large rectal lesion.

**Fig. 1:**
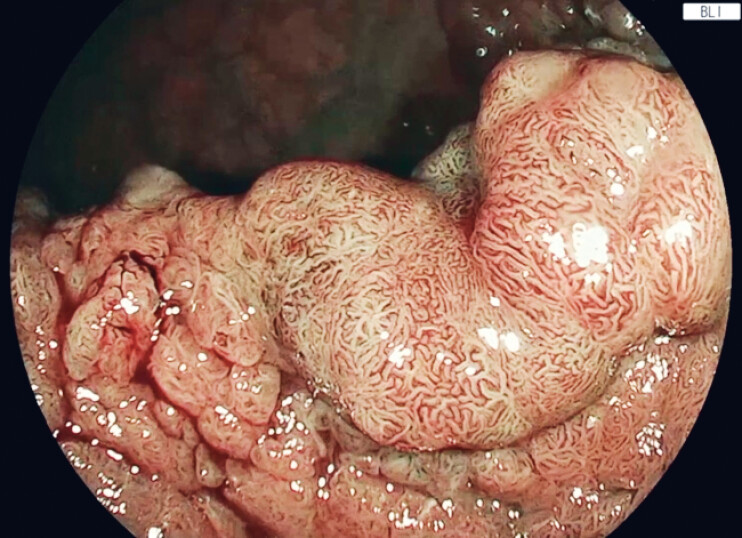
Blue light imaging of the rectal mass.

**Fig. 2:**
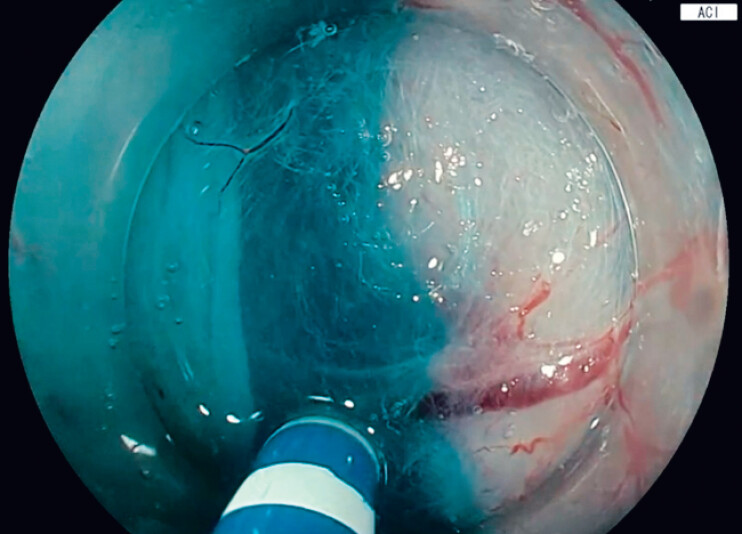
Using amber-red color imaging (ACI) to optimize the visualization of the submucosal plane.

**Fig. 3:**
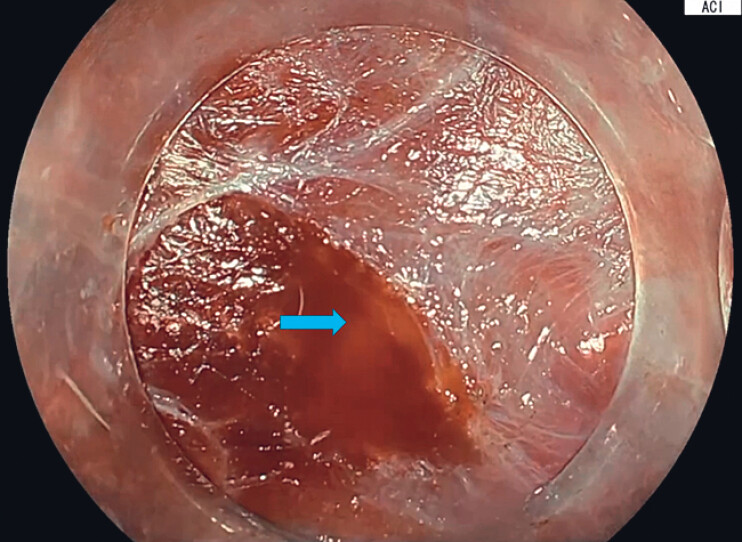
Using amber-red color imaging (ACI) to visualize bleeding blood vessels. The yellow arrow shows the source of bleeding vessels as orange color.

**Fig. 4:**
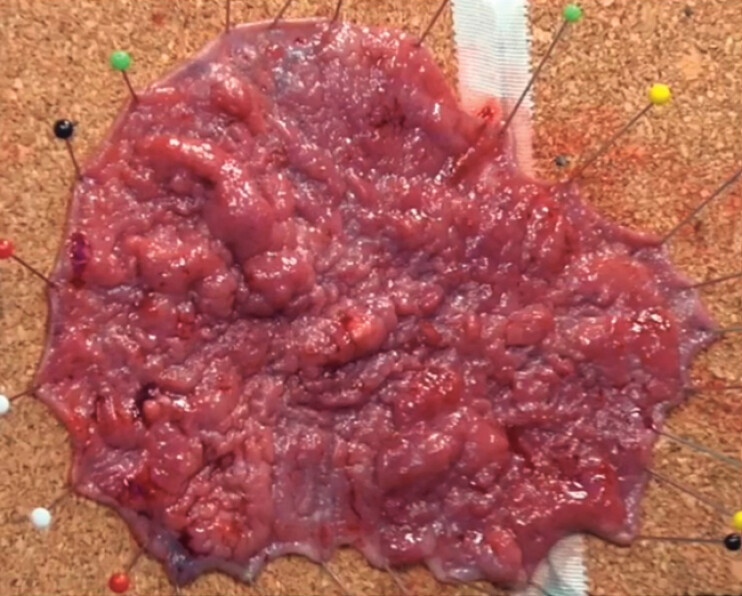
A macroscopic view of rectal mass measuring 11 × 8 cm.

A 66-year-old woman presented with changes in bowel habits. Colonoscopy revealed a 10 cm laterally spreading tumor-granular mixed type, Paris classification 0-IIa, located in the rectum (JNET type 2B), and referred for ESD ( Fig. 1 ). After submucosal injection with 6% hetastarch mixed with 1% epinephrine and indigo carmine, we performed ESD using an ORISE Proknife 1.5 mm (Boston Scientific, MA). Once the initial circumferential incision was completed, two SureTrac (MicroTech, MI) traction systems were applied at the distal border to facilitate two separate submucosal entry points. The traction system is pre-loaded and consists of a endoscopic clip attached to an elastic silicone rubber band with adjustable rings. Submucosal tunneling was then performed by creating two longitudinal tunnels. To facilitate mucosal dissection, ESD was performed using an EG-760Z endoscope with a novel processor EP-8000 (FUJIFILM Co., Tokyo, Japan). A novel image-enhanced technology (FUJIFILM) for ESD, called amber-red color imaging (ACI) was utilized. ACI optimizes the submucosal blue layer and improves the visualization of blood flow in bleeding situations by using amber and orange colors ( Fig. 2 and Fig. 3 ). Under ACI, both tunnels were then connected. To complete the final stages of dissection, two sequential SureTrac traction (MicroTech, MI) devices were applied along the lateral borders of the lesion, and the reminder of dissection was completed ( Fig. 4 ). Following the completion of the ESD, the defect site was assessed revealing no evidence of superficial or deep muscular injury. The total dissection time was approximately 120 minutes. Due to the size of the lesion (11 × 8 cm, ~80% circumferential), the defect was not amenable for endoscopic closure. The patient was observed overnight and discharged the next day. Histopathology revealed well differentiated intramucosal carcinoma with negative deep and horizontal margins and no lymphovascular invasion and was evaluated as a curative resection ( Video 1 ).

Using multi-traction and multi-tunneling techniques with image enhancement for colorectal endoscopic submucosal dissection.Video 1

This case highlights the complementary role of multi-traction and multi-tunneling techniques in facilitating efficient ESD and demonstrates the added value of ACI in enhancing visualization during complex colorectal dissections.

Endoscopy_UCTN_Code_CCL_1AD_2AB

Endoscopy_UCTN_Code_TTT_1AQ_2AD_3AD
